# *Drosophila melanogaster* cellular repressor of E1A-stimulated genes is a lysosomal protein essential for fly development

**DOI:** 10.1016/j.bbamcr.2014.08.012

**Published:** 2014-12

**Authors:** Elisabeth Kowalewski-Nimmerfall, Philipp Schähs, Daniel Maresch, Dubravko Rendic, Helmut Krämer, Lukas Mach

**Affiliations:** a Department of Applied Genetics and Cell Biology, University of Natural Resources and Life Sciences, Muthgasse 18, A-1190 Vienna, Austria; b Department of Chemistry, University of Natural Resources and Life Sciences, Muthgasse 18, A-1190 Vienna, Austria; c Department of Neuroscience, University of Texas Southwestern Medical Center, 6000 Harry Hines Blvd., Dallas, TX 75390-9111, USA; d Department of Cell Biology, University of Texas Southwestern Medical Center, 6000 Harry Hines Blvd., Dallas, TX 75390-9111, USA

**Keywords:** CREG, Cellular repressor of E1A-stimulated genes, dCREG, *D. melanogaster* CREG, GFP, Green fluorescent protein, LERP, Lysosomal enzyme receptor protein, M6P, Mannose 6-phosphate, msCREG, Mouse CREG, PNGase F, Peptide *N*-glycosidase F, Cathepsin, CREG, Lysosome, Mannose 6-phosphate, Protein targeting, Proteolytic maturation

## Abstract

Mammalian cellular repressor of E1A-stimulated genes is a lysosomal glycoprotein implicated in cellular growth and differentiation. The genome of the fruit fly *Drosophila melanogaster* encodes a putative orthologue (dCREG), suggesting evolutionarily conserved physiological functions of this protein. In *D. melanogaster* S2 cells, dCREG was found to localize in lysosomes. Further studies revealed that intracellular dCREG is subject of proteolytic maturation. Processing and turnover could be substantially reduced by RNAi-mediated silencing of cathepsin L. In contrast to mammalian cells, lysosomal delivery of dCREG does not depend on its carbohydrate moiety. Furthermore, depletion of the putative *D. melanogaster* lysosomal sorting receptor lysosomal enzyme receptor protein did not compromise cellular retention of dCREG. We also investigated the developmental consequences of dCREG ablation in whole *D. melanogaster* flies. Ubiquitous depletion of dCREG proved lethal at the late pupal stage once a knock-down efficiency of > 95% was achieved. These results demonstrate that dCREG is essential for proper completion of fly development.

## Highlights

• The lysosomal localization of CREG is evolutionarily conserved.
• Lysosomal delivery of CREG is mediated by different mechanisms in mammals and flies.
• Cathepsin L is the main protease responsible for CREG processing and turnover.
• CREG deficiency causes developmental lethality in *D. melanogaster*.

## 1 Introduction

Cellular repressor of E1A-stimulated genes (CREG) is a mammalian glycoprotein implicated in diverse cellular processes. CREG has been reported to antagonize E1A-mediated transcriptional activation and cellular transformation [1], to inhibit cell division while promoting cell differentiation [2], [3], to regulate the ERK1/2 pathway during cardiac hypertrophy [4], [5], and to protect cells from apoptosis via activation of the PI3K and JNK signaling pathways [6], [7]. However, the molecular mechanisms governing these physiological functions of CREG are still a matter of debate.

The crystal structure of human CREG revealed that CREG monomers display a *β*-barrel fold and that CREG appears to form a homodimeric complex. Its carbohydrate moiety has been shown to contain mannose 6-phosphate (M6P) residues [8]. All three potential *N*-glycosylation sites are mapped on the surface opposite of the dimer interface, and CREG thus potentially represents a multivalent ligand for M6P receptors [9]. Our previous studies demonstrated that murine CREG carries M6P residues at both *N*-glycosylation sites, and that both M6P receptors are involved in its intracellular retention. In murine fibroblasts, CREG is residing in lysosomal compartments and is proteolytically processed due to the action of lysosomal cysteine proteinases [10]. Interestingly, the genome of the fruit fly *Drosophila melanogaster* encodes a putative orthologue of CREG. Like its human counterpart, *D. melanogaster* CREG (dCREG) shares limited sequence homology to adenoviral E1A and binds *in vitro* to *D. melanogaster* retinoblastoma protein [1]. However, its biochemical properties and subcellular localization in *D. melanogaster* cells have not been investigated as yet.

In mammals, the intracellular sorting pathways for newly synthesized lysosomal proteins are well understood and depend mostly on the action of two M6P receptors: the M6P/insulin-like growth factor II receptor (M6P/IGF2R) and the 46-kDa M6P receptor (MPR46). In the *trans*-Golgi network (TGN), these receptors bind proteins carrying *N*-glycans modified with M6P groups. The receptors are then concentrated in membrane buds via interaction of their cytoplasmic tails with adaptor proteins like the heterotetrameric adaptor protein (AP) complexes and the monomeric Golgi-localizing, γ-ear-containing, ARF-binding proteins (GGAs). These complexes are then concentrated in clathrin-coated vesicles which then bud from the TGN, carrying the cargo proteins to endosomal compartments [11], [12], [13].

Biosynthetic sorting of lysosomal enzymes in *D. melanogaster* cells is far less understood. Protein transport to *D. melanogaster* lysosomes appears to be independent of the M6P recognition marker, although a putative phosphotransferase homologue has been identified in the fly genome [14]. Interestingly, recent studies have identified a *D. melanogaster* protein, lysosomal enzyme receptor protein (LERP), which is closely related to M6P/IGF2R. The luminal domain of LERP consists of five repeating domains, which contain potential carbohydrate-binding regions. However, it has been shown that LERP is unable to bind M6P residues [15]. Recently, it was demonstrated that intracellular trafficking of LERP in *D. melanogaster* cells is dependent on GGA, and that LERP depletion leads to decreased processing of lysosomal cathepsin L [16]. Furthermore, LERP expression was found to rescue the missorting of lysosomal proteins in M6P receptor-deficient mouse fibroblasts [15]. However, direct interaction between LERP and lysosomal proteins has not been observed so far.

In this study, we report a detailed characterization of the biosynthesis and subcellular localization of dCREG in *D. melanogaster* S2 cells. Furthermore, the impact of LERP on the intracellular transport and proteolytic maturation of dCREG was investigated. Finally, we examined the consequences of RNAi-mediated knock-down of dCREG in flies.

## 2 Materials and methods

### 2.1 Antibodies

Recombinant dCREG and LERP produced in insect cells were purified as described below and then used to immunize rabbits (Gramsch Laboratories, Schwabenhausen, Germany). Antibodies were purified by affinity chromatography with immobilized recombinant proteins as reported [10], [17], using 0.1 M glycine/HCl buffer (pH 2.3) for elution. Recombinant *D. melanogaster* Golgi α-mannosidase II [18] was kindly provided by Douglas Kuntz (University of Toronto, Canada) and used to prepare antibodies in mice. Mouse monoclonal antibodies to α-tubulin (Sigma-Aldrich), insect cathepsin L (R&D Systems, Minneapolis, MN) and the V5 epitope (Invitrogen, Carlsbad, CA) were purchased from commercial suppliers.

### 2.2 Construction of pMT vectors

All oligonucleotide primers used in this study were custom-synthesized by Sigma-Aldrich (St. Louis, MO), and restriction enzymes were purchased from Fermentas (St. Leon-Rot, Germany). For amplification of the dCREG coding sequence, total RNA was extracted from *D. melanogaster* S2 cells using the RNeasy kit (Qiagen, Venlo, Netherlands) according to the instructions of the supplier. dCREG cDNA was then generated using the OmniScript kit (Qiagen) with the following primers: 5′-ATGAAAACCTTTCACTCCCTACTATTC-3′ (sense) and 5′-TCAATTCGAAACAGCGTAATAGTCAG-3′ (antisense). The resulting 636-bp PCR fragment was then cloned into pMT/V5-His-TOPO (Invitrogen), yielding pMT/dCREG. The dCREG cDNA was also cloned into the *Kpn*I and *Xho*I sites of pMT/V5His after amplification with 5′-TTGGGTACCATGAAAACCTTTCACTCCCTACTATCC-3′ (sense) and 5′-TTACTCGAGATTCGAAACAGCGTAATAGTCAG-3′ (antisense), resulting in pMT/dCREG-V5His. Construction of the dCREG-GFP vector was performed by transferring the GFP sequence from pEGFP-N3 (BD Biosciences, Erembodegem, Belgium) into the *Not*I and *Sac*II sites of pMT/dCREG-V5His, giving rise to pMT/dCREG-GFP-V5His. To mutate the single *N*-glycosylation site of dCREG (Asn^87^) into Gln, the Quick-Change site-directed mutagenesis kit (Stratagene, La Jolla, CA) was used with 5′-AGCGATGCTAATCAAAGGTCCACTGGAC-3′ (sense) and 5′-GTCCAGTGGACCTTTGATTAGCATCGCT-3′ (antisense) as primers.

### 2.3 Construction of other expression vectors

A cDNA fragment encoding dCREG lacking its signal peptide was obtained by PCR using the primers 5′-AGCTCTAGATACTCCCGCCGGAAAGATG-3′ (sense) and 5′-GTAGGTACCTCAATTCGAAACAGCGTAATAGTCAG-3′ (antisense). The PCR product was cleaved with *Xba*I and *Kpn*I at the underlined sites and ligated into pVTBacHis-1 baculovirus transfer vector [19], digested with the same enzymes. In this construct, the recombinant protein is placed downstream of the melittin signal peptide, a hexahistidine sequence and an Xpress epitope (Asp-Leu-Tyr-Asp-Asp-Asp-Asp-Lys). To replace the latter by a FLAG epitope (Asp-Tyr-Lys-Asp-Asp-Asp-Asp-Lys), pVTBacHis-1 was subjected to site-directed mutagenesis with the primers 5′-GACAGCAAATGGGTCGGGATTACAAGGACGATGACGATAAGTC-3′ (sense) and 5′-GACTTATCGTCATCGTCCTTGTAATCCCGACCCATTTGCTGTC-3′ (antisense), giving rise to pVT-BacHis-FLAG. A cDNA fragment encoding the LERP ectodomain (amino acids 42–781) was amplified using the primers 5′-AATCTAGAGACGCAGCCAATCAGC-3′ (sense) and 5′-AATCTAGATCAGAGGGAAAGGAACTCGC-3′ (antisense), cleaved with *Xba*I at the underlined sites and then ligated into pVTBacHis-FLAG. To generate the expression plasmid pFO4/dCREG, amplification of a cDNA encoding a truncated version of dCREG lacking the N-terminal signal peptide was achieved by PCR using the primers 5′-CACAGATCTTACTCCCGCCG-3′ (sense) and 5′-GCAGAATTCTCAATTCGAAACAGCG-3′ (antisense). The corresponding mouse CREG cDNA fragment was obtained with the primers 5′-AAAGGATCCCGCGGAGGCCGGGACCACG-3′ (sense) and 5′-CCCGAATTCTCACTGCAGCGTGACGTTAAAATATTCTTCAG-3′ (antisense). The PCR products were digested with *Eco*RI and *Bgl*II or *Bam*HI and then ligated into the *EcoR*I and *BamH*I sites of pFO4/hCREG (kindly provided by Michael Sacher, Biotechnology Research Institute, Montreal, Canada). pFO4/dCREG has an open reading frame consisting of a short leader sequence (Met-Gly-Ser-Ser-His-His-His-His-His-His-Gly-Ser) followed by dCREG residues 24–212, while pFO4/msCREG encodes amino acids 32–220 of mouse CREG fused to the same N-terminal tag.

### 2.4 Cell culture and transfection

*D. melanogaster* S2 cells (Invitrogen) were maintained in Schneider's insect medium supplemented with 10% FBS, 50 units/ml penicillin and 50 μg/ml streptomycin at 27 °C. S2 cells were transfected using the *Drosophila* Expression System (Invitrogen) with a 30:1 ratio of the pMT/V5-His expression constructs and pCoBlast (Invitrogen). Stably transfected cells were obtained by selection with 30 μg/ml blasticidin (Invitrogen). Blasticidin-resistant clones were obtained after 10–14 days and propagated in the presence of 10 μg/ml blasticidin.

### 2.5 Gene silencing experiments in S2 cells

For synthesis of target-specific double-stranded RNA, fragments were amplified by PCR with gene-specific primers flanked by T7 promoter sites at the 5′ end. The resulting fragments were used as templates for *in vitro* transcription performed with the HiScribe RNAi Transcription Kit (New England Biolabs, Beverly, MA) following the manufacturer's instructions. The following gene-specific primer sequences were used for double-stranded RNA preparation: 5′-CACCAAGTACGGCAACAATG-3′ (cathepsin L, sense); 5′-TAGACGCCCTCCGAGTAGAA-3′ (cathepsin L, antisense); 5′-GATGCAAGGCAACCACCTAT-3′ (LERP, sense); 5′-CGGCACAGCCTAATTGGTAT-3′ (LERP, antisense). S2-dCREG cells were seeded at a density of 2 × 10^6^ cells in 1 ml serum-free medium, to which 15 μg double-stranded RNA was added. After 60 min, 1 ml serum-containing medium was added to the cells prior to incubation for 72 h at 27 °C.

### 2.6 Heterologous expression of dCREG and LERP in insect cells

*Spodoptera frugiperda* Sf9 and Sf21 cells (both obtained from the American Type Culture Collection, Manassas, VA) were grown in IPL-41 medium containing 5% heat-inactivated FBS, 50 units/ml penicillin and 50 μg/ml streptomycin at 27 °C. pVTBacHis-1/dCREG or pVTBacHis-FLAG/LERP (1 μg) was cotransfected with 200 ng BaculoGold viral DNA (BD Biosciences) into Sf9 cells using Lipofectin (Invitrogen) as recommended by the manufacturer. Supernatant containing recombinant baculovirus was harvested after 5 days and used for infection of Sf21 cells.

### 2.7 Purification of recombinant proteins produced in insect cells

Culture supernatants (200 ml) of Sf21 cells infected with recombinant baculovirus were cleared by centrifugation and dialyzed two times against 2 L of dialysis buffer (10 mM sodium phosphate buffer, pH 7.0, 40 mM NaCl, 0.02% NaN_3_). The supernatant was supplemented with 20 mM imidazole and 10% (v/v) glycerol and then loaded on a 5 ml column of Chelating Sepharose (GE Healthcare, Little Chalfont, United Kingdom) charged with Ni^2 +^ ions, equilibrated in the same buffer. After successive washes with 40 mM and 80 mM imidazole, the recombinant protein was eluted with 250 mM imidazole in dialysis buffer. Protein-containing eluate fractions were pooled and dialyzed twice against 1 L of PBS containing 0.02% (w/v) NaN_3_ prior to concentration by ultrafiltration and addition of proteinase inhibitors (1 mM PMSF, 5 μg/ml E-64 and 5 μg/ml leupeptin). Recombinant LERP was further purified by loading on a 1 ml Anti-FLAG M2 affinity column (Sigma-Aldrich). The column was washed with 10 ml PBS containing 0.02% NaN_3_. Bound LERP was then eluted with 5 ml 100 μg/ml FLAG peptide in the same buffer. The eluate fractions were pooled, dialyzed and then concentrated as above.

### 2.8 Purification of recombinant proteins produced in *Escherichia coli*

*E. coli* BL21 (DE3) cells (Invitrogen) transformed with pFO4/CREG were grown in 1 L of LB medium containing 100 μg/ml ampicillin at 37 °C until an optical density at 600 nm of 0.6 was reached. Recombinant protein production was then induced by addition of 1 mM IPTG. After induction, the cells were cultured for 16 h at 30 °C. The bacterial cells were harvested by centrifugation, resuspended in 20 ml lysis buffer (50 mM Tris/HCl, 1 mM PMSF, pH 8.0), and disintegrated by ultrasonication. After centrifugation of the lysates for 20 min at 15,000 ×*g* and 4 °C, the supernatant was adjusted to 0.3 M NaCl, 10 mM imidazole and 5% (v/v) glycerol, and loaded onto a 1 ml column of His-Select beads (Sigma-Aldrich), equilibrated in the same buffer without glycerol. After extensive washing with equilibration buffer, the recombinant protein was eluted with 250 mM imidazole in equilibration buffer. Protein-containing eluate fractions were pooled and dialyzed twice against 1 L of PBS containing 0.02% (w/v) NaN_3_.

### 2.9 Size exclusion chromatography

The quaternary structure of recombinant CREG was examined by size exclusion chromatography on a Superdex 75 HR 10/30 column (GE Healthcare) equilibrated with 50 mM sodium phosphate buffer (pH 7.0) containing 150 mM NaCl, using an ÄKTA purifier system (GE Healthcare). Elution was performed with a flow rate of 0.5 ml/min, and the absorbance of the eluate was recorded at 280 nm.

### 2.10 Circular dichroism spectroscopy

Circular dichroism spectroscopy was performed at 25 °C on a PiStar-180 spectrometer (Applied Photophysics, Leatherhead, United Kingdom). The instrument was flushed with nitrogen at a flow rate of 5 L per min. For recording of far UV spectra (180–260 nm), recombinant proteins dissolved in 5 mM sodium phosphate buffer (pH 7.0) were brought to identical concentrations as determined by measuring the optical density of the solutions at 280 nm. Each spectrum was automatically corrected for the birefringence of the cell.

### 2.11 *In vitro* processing of CREG by cathepsins B and L

*In vitro* processing assays were carried out in 10 μl 50 mM sodium acetate buffer (pH 5.5) containing 2 mM cysteine. 100 ng recombinant human cathepsin B [20] or cathepsin L (kindly provided by John Mort, Shriners Hospital for Children, Montreal, Canada) were added to 2 μg recombinant mouse CREG or dCREG and incubated for 30 min to 16 h at 37 °C. The reaction was stopped by addition of SDS-PAGE sample buffer containing 50 μg/ml E-64 and subsequent heating for 5 min at 95 °C.

### 2.12 Cleavage site analysis

N-terminal sequence analysis of bands blotted onto PVDF membranes (Bio-Rad) as described previously [21] was performed by Edman degradation on an Applied Biosystems Procise 492 protein sequencer (Protein Micro-Analysis Facility, Medical University of Innsbruck, Austria). dCREG treated with cathepsin L was also characterized by LC-electrospray ionization-MS, either as intact protein or after digestion with trypsin or chymotrypsin subsequent to separation by SDS-PAGE [22]. The samples were fractionated by nano-LC (150 × 0.32 mm BioBasic-18, Thermo Scientific, Waltham, MA) using a gradient of 1–80% acetonitrile. On-line data acquisition was conducted in positive-ion mode on a maXis-4GQ-TOF mass spectrometer (Bruker, Billerica, MA). MS2 scans of dominant precursor peaks were manually analyzed with Data Analysis software version 4.0 (Bruker).

### 2.13 Preparation of microsomal extracts

1 × 10^8^ S2-dCREG cells were harvested with a cell scraper, washed with PBS, resuspended in 1 ml 3 mM imidazole buffer (pH 7.4) containing 0.25 M sucrose and lysed by 20 strokes with a tight-fitting pestle using a Dounce homogenizer. Postnuclear supernatants were obtained by low-speed centrifugation (5 min at 400 ×*g*) and then centrifuged for 60 min at 105,000 ×*g* and 4 °C. The pellets were extracted for 30 min at 0 °C with 250 μl 50 mM Tris/HCl (pH 7.4), 150 mM NaCl, 1 mM PMSF, 5 μg/ml leupeptin, 5 μg/ml E-64 and 0.1% Triton X-100. The samples were then cleared by centrifugation for 10 min at 15,000 ×*g* prior to further analysis.

### 2.14 Secretion studies

2 × 10^6^ S2-dCREG cells were washed with PBS and then incubated for 72 h at 27 °C in 2 ml of culture medium containing 0.5 mM CuSO_4_. The conditioned media were then aspirated and cleared by low-speed centrifugation (5 min, 320 ×*g*) and subsequent passage through 0.22-μm filters. After washing with PBS and harvesting by scraping, cells were sonicated (3 × 10 s) in 250 μl 50 mM Tris/HCl (pH 7.4) containing 150 mM NaCl, 1 mM PMSF, 5 μg/ml leupeptin and 5 μg/ml E-64. Prior to extraction at 0 °C for 30 min, Triton X-100 was added to a final concentration of 1%. Samples were cleared by centrifugation for 10 min at 15,000 ×*g* and subjected to SDS-PAGE and immunoblotting analysis.

### 2.15 Fluorescence microscopy

S2 cells expressing dCREG-GFP were plated on acid-washed glass coverslips treated with 0.5 mg/ml concanavalin A (Sigma-Aldrich) and then induced for 16 h with 0.5 mM CuSO_4_ prior to fixation by incubation for 10 min in 4% paraformaldehyde in PBS. After blocking with PBS containing 0.2% BSA or 3% normal goat serum in PBS for 1 h, the cells were incubated for 1 h with monoclonal anti-insect cathepsin L (5 μg/ml) or polyclonal mouse antiserum to *D. melanogaster* Golgi-mannosidase II (1:500) diluted with PBS containing 0.2% BSA and 0.1% saponin. After a second blocking step in PBS containing 0.1% saponin and 5% FBS (1 h), bound primary antibodies were detected by incubation for 1 h with Cy3-conjugated affinity-purified secondary antibodies (Jackson ImmunoResearch, West Grove, PA) used at a concentration of 5 μg/ml in PBS containing 5% FBS and 0.1% saponin. All steps were performed at room temperature. The immunostained cells were examined with a Leica TCS SP5 confocal microscope equipped with Ar and He/Ne lasers. Images from the confocal system were deconvoluted using Huygens Essential 3.7 software (Scientific Volume Imaging, Hilversum, Netherlands) and then imported into Adobe Photoshop CS 8.0 for coloration. For staining of acidic organelles, S2 cells stably expressing dCREG-GFP were induced as above and then incubated in medium supplemented with 100 nM LysoTracker Red DND-99 (Molecular Probes, Eugene, OR). Cells were incubated for 30 min at 27 °C, then briefly rinsed with PBS and immediately observed by confocal laser-scanning microscopy. Lysotracker staining of fat bodies isolated from timed third-instar larvae (age: 92 h) was done as above for 5 min at room temperature prior to confocal imaging of 5–10 tissue areas per sample.

### 2.16 Maintenance of fly strains

The following lines were used: Canton-S, Oregon-R, w^1118^ (wild-type); the double-balancer If/Cyo; Sb/TM3, Ser; the ubiquitously expressed driver da-GAL4 (Bloomington Stock Center, Bloomington, IN); UAS-CREG-RNAi^KK^ (line: 108999), UAS-cathepsin L-RNAi^KK^ (110619), UAS-cathepsin B-RNAi^KK^ (108315), VDRC (Vienna, Austria); Lsp2-GAL4-UAS-GFP-Atg8 [23]. Animals were reared at 18 °C or 25 °C on regular fly food (10% corn meal, 2.25% dried yeast, 1.25% soya flour, 2.75% molasses, 10% malt extract, 1.05% agar, 0.8% propionic acid and 0.175% nipagin A). Crosses were performed by standard procedures.

### 2.17 Generation of UAS-dCREG-GFP flies

dCREG-GFP-V5His cDNA was amplified by PCR from the pMT/dCREG-GFP-V5His vector using the following primers: 5′-TAAGAATTCATGAAAACCTTTCACTCCCTAC-3′ (sense) and 5′-AAGACTAGTCTAATGGTGATGGTGATGATG-3′ (antisense). The resulting PCR product was then cloned into the *Eco*RI and *Spe*I sites (underlined) of pUASTattB (provided by Peter Duchek, Institute of Molecular Biotechnology, Vienna, Austria). Transgenic flies were then generated using a ΦC31-based integration system, leading to targeted insertion of the ectopic sequence on the third chromosome (VDRC, Vienna, Austria).

### 2.18 Preparation of fly extracts

Freshly eclosed flies were resuspended in 50 μl extraction buffer (50 mM Tris/HCl (pH 7.4), 150 mM NaCl, 1 mM PMSF, 5 μg/ml leupeptin, 5 μg/ml E-64 and 1% Triton X-100) per fly and then thoroughly ground with a micropestle. The samples were then placed on ice for 30 min and subsequently cleared by centrifugation for 10 min at 15,000 ×*g* and 4 °C.

### 2.19 Temperature-shift experiments

w^1118^ and UAS-CREG-RNAi; da-GAL4 flies were reared at 18 °C, put on fresh medium and then shifted to 25 °C. Parents were removed from the vials after 5 days at the latest. Eclosed flies were counted as well as late pupae that did not hatch even after 20 days of cultivation. As a control, w^1118^ and UAS-CREG-RNAi; da-GAL4 flies were kept at 18 °C and counted as above. To account for the longer life cycle at 18 °C, numbers of flies and pupae were recorded for up to 6 weeks. In total, 600–1000 flies and pupae were counted for every condition.

### 2.20 Other methods

Enzymatic deglycosylation of proteins, SDS-PAGE, immunoblotting and mass spectrometric *N*-glycan analysis were performed as previously described [10]. Total protein content was determined either with the Bio-Rad protein assay kit or the BCA protein assay kit (Pierce, Rockford, IL) as appropriate, using BSA as a standard. Statistical analyses were performed using Student's *t*-test, with *p* < 0.05 being considered significant.

## 3 Results

### 3.1 dCREG is posttranslationally modified by *N*-glycosylation and limited proteolysis

Human CREG is a homodimeric protein [9]. Elucidation of the quaternary status of dCREG produced in *E. coli* by means of size exclusion chromatography revealed that, like human CREG, the insect protein is a dimer with an approximate native molecular mass of 41 kDa. Recombinant dCREG was also subjected to an evaluation of its structural features by circular dichroism spectroscopy. dCREG shows the typical properties of a *β*-sheet protein combined with *α*-helical elements and thus displays a similar secondary structure as human CREG (data not shown).

**Fig. 1:**
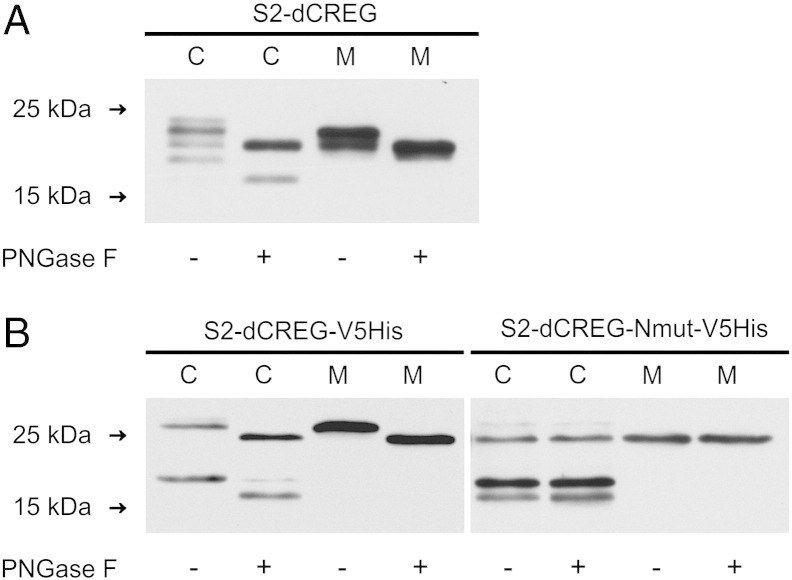
Posttranslational modification of dCREG by proteolytic maturation and *N*-glycosylation. (A) *D. melanogaster* S2 cells overexpressing dCREG under the control of the metallothionein promoter (S2-dCREG) were incubated for 72 h in the presence of 0.5 mM CuSO_4_. Cell lysates (C; 6 μg) and media (M) corresponding to 0.15 μg cellular protein were treated for 1 h in the presence (+) or absence (−) of PNGase F. Proteins were then subjected to immunoblotting analysis with antibodies to dCREG. (B) S2 cells expressing V5His-tagged dCREG (S2-dCREG-V5His) and S2 cells expressing V5His-tagged, *N*-glycosylation-minus dCREG (S2-dCREG-Nmut-V5His) were incubated for 72 h in the presence of 0.5 mM CuSO_4_. Cell extracts (C; 6 μg) and conditioned media (M) corresponding to 0.15 μg cellular protein were incubated with (+) or without (−) PNGase F as outlined above, immunoblotted and probed with antibodies to dCREG. The migration positions of selected molecular mass standards are indicated. The results shown are representative of three independent experiments.

Recombinant dCREG produced in the baculovirus expression system was used to raise a rabbit antiserum. Monospecific antibodies were then isolated with the aid of immobilized dCREG made in *E. coli*. Immunoblot analysis of *D. melanogaster* S2 cells revealed that endogenous dCREG was hardly detectable in crude lysates. For further studies, we therefore decided to overexpress dCREG cDNA in S2 cells under the control of an inducible promoter to minimize overloading of the endogenous sorting machinery. This approach allowed the detection of intracellular dCREG as four bands ranging from 19 to 23 kDa, whereas two bands of 20 and 22 kDa were observed for the secreted fraction of the protein (Fig. 1A).

Human and mouse CREG are *N*-glycosylated [2], [10], and dCREG contains one potential *N*-glycosylation site at position Asn^87^. To examine if the *D. melanogaster* protein is indeed posttranslationally modified with *N*-glycans, microsomal extracts and conditioned media of S2-dCREG cells were treated with peptide *N*-glycosidase F (PNGase F). Digestion with PNGase F resulted in a reduction of the apparent molecular mass of both cellular and secreted dCREG (Fig. 1A), verifying that the protein indeed carries *N*-linked oligosaccharides. Deglycosylation reduced the apparent molecular mass of the secreted 22-kDa band to 20 kDa, thus indicating that the two dCREG variants present in the culture medium correspond to the glycosylated and carbohydrate-free forms of the same polypeptide. Analysis of the amino acid sequence of dCREG with SignalP 4.0 [24] identified the first 23 amino acids as potential N-terminal signal sequence for targeting to the secretory pathway. Hence, the predicted molecular mass of dCREG is 21.6 kDa, which is in reasonable agreement with our experimental data. The bands of 20 and 22 kDa corresponding to the secreted forms of dCREG were also present in cell extracts, in addition to two other variants of 19 and 23 kDa. After PNGase F treatment, the 19-kDa band migrated as a 17-kDa polypeptide while the molecular mass of the 23-kDa form was reduced to 20 kDa (Fig. 1A). The detection of the 17-kDa polypeptide after enzymatic deglycosylation suggests that intracellular dCREG is not only processed by signal peptidase but also subject of additional N-terminal proteolytic maturation like its human and mouse orthologues [10], [25]. For the 23-kDa band, conversion into the 20-kDa polypeptide was also observed upon digestion with endoglucosaminidase H, whereas the other forms of dCREG were not sensitive to this more restrictive endoglycosidase (data not shown). This indicates that 23-kDa dCREG carries high-mannose *N*-glycan precursors and thus most likely represents a biosynthetic intermediate located in the endoplasmic reticulum. In contrast, the dCREG species of 19 and 22 kDa are modified with fully processed paucimannosidic oligosaccharide structures as typical for glycoproteins that have passed through the insect Golgi apparatus [26]. The exclusive presence of paucimannosidic *N*-glycans on secreted dCREG was verified by mass spectrometric analysis of the purified recombinant protein produced in insect cells (data not shown).

To analyze if *N*-glycosylation of dCREG is important for intracellular retention and biosynthetic sorting of the protein, we prepared a glycosylation-minus variant by replacing Asn^87^ with Gln by site-directed mutagenesis. S2-dCREG-V5His and S2-dCREG-Nmut-V5His cells (stably expressing wild-type or Asn^87^ → Gln dCREG, both modified with a C-terminal V5His-tag) were generated and then analyzed by immunoblotting. Interestingly, dCREG-Nmut-V5His was still retained by the cells and also subject of proteolytic processing. Despite overexpression of the mutant protein which could cause excessive secretion, the extent of its intracellular retention was 5.5 ± 1.3%, which was even higher than for wild-type dCREG-V5His (1.1 ± 0.1%; p = 0.029). Treatment with PNGase F shifted the 19-kDa and 27-kDa bands of wild-type dCREG-V5His to their unglycosylated counterparts of 17 and 25 kDa, whereas bands of dCREG-Nmut-V5His were, as expected, not sensitive to PNGase F treatment and co-migrated with the deglycosylated wild-type proteins (Fig. 1B). In addition, dCREG-Nmut-V5His displayed a prominent 19-kDa band which was barely detectable in wild-type dCREG-V5His treated with PNGase F. Like the 25-kDa full-length protein, this 19-kDa polypeptide reacted with anti-V5 antibodies, whereas the wild-type and mutant 17-kDa forms failed to do so (data not shown). This suggests that the 19-kDa variant of dCREG-Nmut-V5His is a processing intermediate which has undergone N-terminal proteolytic maturation but still carries the C-terminal V5His-tag. While C-terminal processing of intracellular dCREG-Nmut-V5His was less efficient than in the case of its wild-type counterpart, removal of the N-terminal propeptide (73 ± 2%) was even more pronounced than for the wild-type protein (42 ± 3%; p = 0.001). Collectively, these data indicate that the presence of an *N*-glycan is dispensable for transport of dCREG to its intracellular destination and subsequent proteolytic processing of the protein in this compartment.

### 3.2 dCREG can be processed by lysosomal cysteine proteinases

**Fig. 2:**
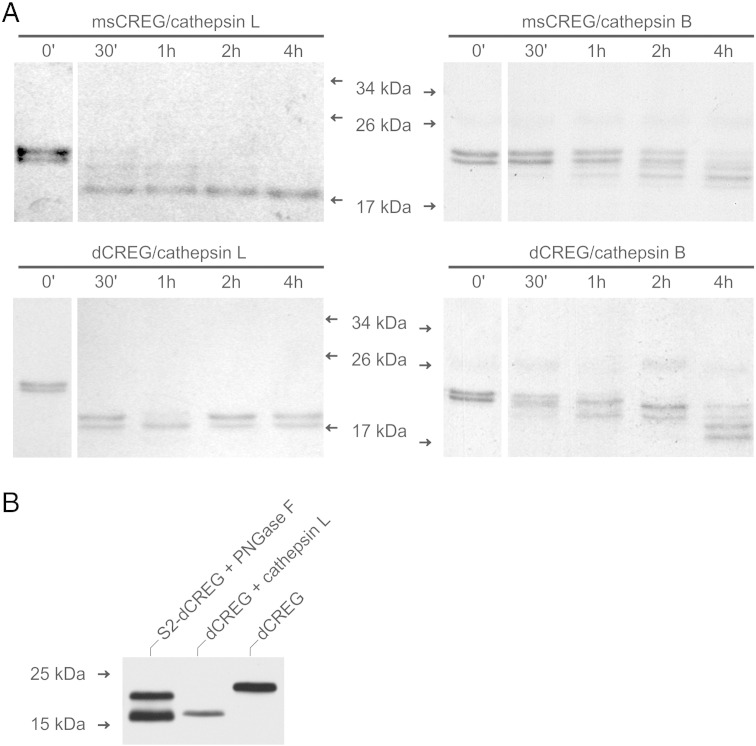
CREG can be proteolytically processed by lysosomal cysteine proteinases. (A) Recombinant mouse CREG (msCREG) and dCREG were incubated with cathepsins B or L for up to 4 h. Samples were then analyzed by SDS-PAGE followed by Coomassie Brilliant Blue staining. (B) Microsomal extracts of S2-dCREG cells (10 μg) were deglycosylated with PNGase F. N-terminally tagged dCREG produced in *E. coli* (30 ng) was subjected to *in vitro* processing by cathepsin L for 4 h. The samples were separated by SDS-PAGE and analyzed by immunoblotting with antibodies to dCREG. The migration positions of selected molecular mass standards are indicated.

Our previously published studies revealed that maturation of CREG in murine fibroblasts involves the action of lysosomal cysteine cathepsins [10]. Four of these enzymes (cathepsins B, F, L and K) have been identified in S2 cells by a functional proteomics screen [27]. Recently, Eissenberg et al. [16] showed that cathepsin L (74%) and cathepsin B (21%) account together for more than 90% of the cysteine proteinase activity of S2 cells. To investigate if cathepsins B and L are capable of processing CREG, we performed *in vitro* processing assays using recombinant mouse CREG (msCREG) and dCREG as substrates. Both CREG proteins could be processed by either enzyme, leading to a final processing product of about 18 kDa (msCREG) and 17 kDa (dCREG), respectively. In both cases, cathepsin L was much faster in processing than cathepsin B (Fig. 2A). This is in good agreement with cathepsin L being a far more potent endopeptidase than cathepsin B which primarily acts as a dipeptidyl carboxypeptidase [28]. For comparison of dCREG processed by cathepsin L *in vitro* with the endogenous forms of the protein occurring in S2 cells, immunoblotting analysis of *in vitro* digested dCREG and deglycosylated extracts of S2-dCREG cells was performed. This revealed that the cathepsin L cleavage product co-migrates with the mature intracellular protein (Fig. 2B).

Given this evidence that cathepsin L could be involved in dCREG processing *in vivo*, the N-terminal sequence of recombinant dCREG incubated with cathepsin L *in vitro* was determined by Edman degradation. The processed protein was found to be missing its 14 N-terminal residues. Since cathepsin L is strictly acting as an endopeptidase [28], it can be inferred that the K^14^–R^15^ bond is preferentially cleaved (EYK^14^↓R^15^EQEL). This was confirmed by MS analysis, which also revealed minor amounts of further truncated sequences starting with E^16^, Q^17^ and E^18^. Interestingly, the N-terminal processing site corresponds closely to that of cathepsin L in msCREG (DVD^12^↓R^13^RLPP) and to the authentic N-terminus of mature human CREG (DEASR^14^↓L^15^PP [25]). MS analysis also demonstrated that recombinant dCREG does not undergo C-terminal processing when exposed to cathepsin L (data not shown). This suggests that the same applies to the mature protein accumulating within cells, which is in good agreement with our previous findings for endogenous CREG in mouse fibroblasts [10].

### 3.3 Cathepsin L is involved in dCREG turnover and processing *in vivo*

**Fig. 3:**
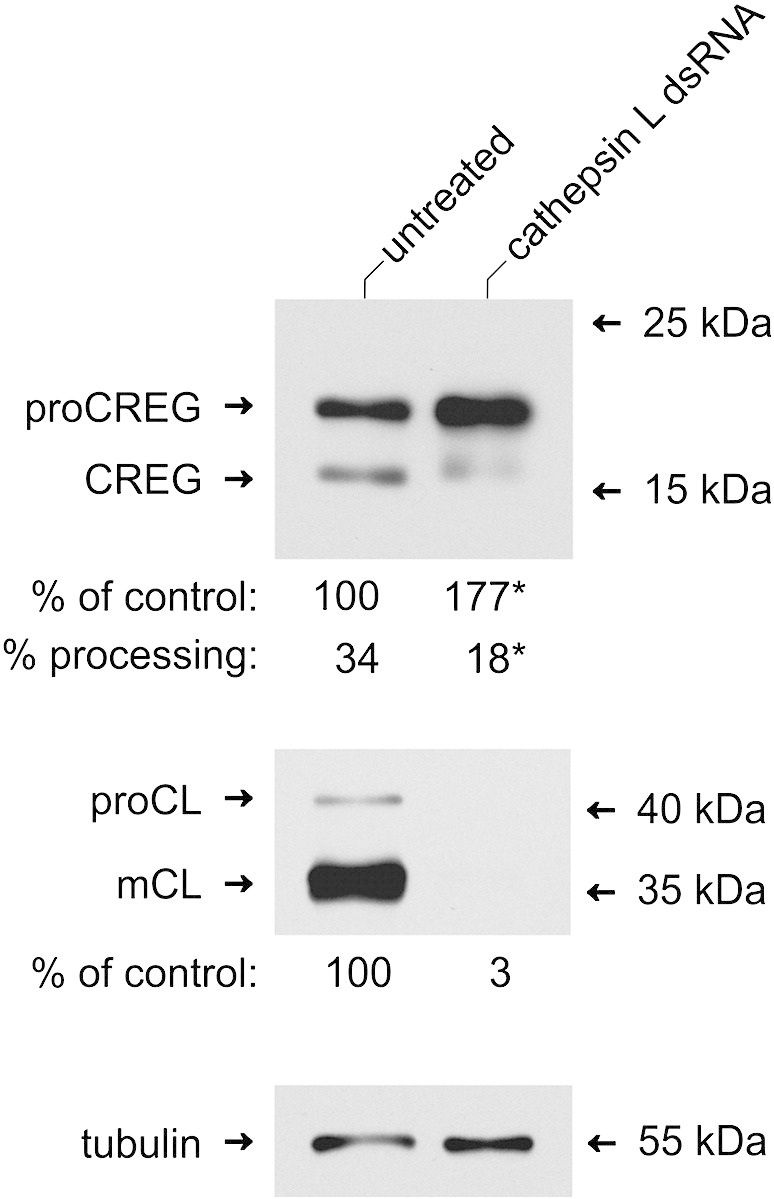
Effects of RNAi-mediated knock-down of cathepsin L on dCREG processing and turnover in S2 cells. S2-dCREG cells were induced with 0.5 mM CuSO_4_ and incubated with double-stranded (ds) cathepsin L RNA for 72 h. Untreated cells cultivated under the same conditions were used as controls. Cell lysates (6 μg) were deglycosylated with PNGase F and subjected to SDS-PAGE. Immunoblotting was then performed with antibodies to dCREG and cathepsin L. Tubulin served as loading control. Band intensities were determined by densitometry. Data are expressed as means of 3 independent experiments. The migration positions of selected molecular mass standards are indicated. proCREG, full-length dCREG; CREG, processed dCREG; proCL, procathepsin L; mCL, mature cathepsin L. *p ≤ 0.001 (comparison of knock-down cells and controls).

Intracellular processing of dCREG was further analyzed using RNAi-mediated depletion of cathepsin L in S2 cells. The knock-down efficiency was determined by immunoblotting. Only 3 ± 1% residual immunoreactive cathepsin L could be detected after the double-stranded RNA treatment (Fig. 3). This almost quantitative removal of cathepsin L led to a 1.8 ± 0.1-fold higher dCREG accumulation (p < 0.001) in S2-dCREG cells and significantly reduced the extent of intracellular dCREG processing from 34 ± 2% to 18 ± 1% (p = 0.001). Although these results clearly show that endogenous cathepsin L is involved in turnover and processing of dCREG, other cellular proteinases are apparently also engaged in the latter process. Similar observations were made for the proteolytic maturation of endogenous CREG in mouse fibroblasts [10].

### 3.4 Intracellular dCREG is a lysosomal protein

**Fig. 4:**
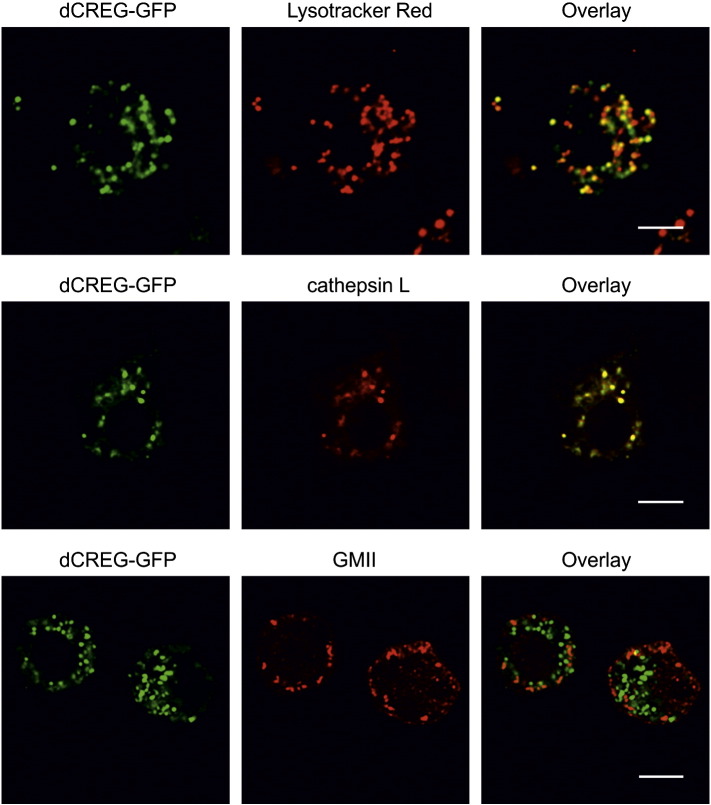
dCREG co-localizes with lysosomal markers. S2 cells stably transfected with a cDNA construct encoding GFP fused to the C-terminus of dCREG (dCREG-GFP) were stained with Lysotracker Red and analyzed by live-cell imaging (top). S2 cells expressing dCREG-GFP were fixed, permeabilized and incubated with antibodies to cathepsin L (middle) or Golgi-mannosidase II (GMII; bottom). Bound antibodies were then detected with Cy3-labeled secondary antibodies followed by confocal laser-scanning microscopy. Co-localization was assessed by merging of the individual images. Bars, 10 μm.

To assess the subcellular localization of dCREG in S2 cells, confocal fluorescence microscopy was performed. S2 cells were stably transfected with a cDNA construct encoding a chimeric protein consisting of GFP fused to the C-terminus of dCREG (dCREG-GFP). dCREG-GFP was detected in vesicles dispersed throughout the cytoplasm, a subcellular distribution reminiscent of endosomes and lysosomes. Live-cell staining of S2-dCREG-GFP cells with Lysotracker Red showed that a substantial fraction of dCREG-GFP is located in acidic compartments. Moreover, double-labeling experiments revealed a large extent of co-localization between dCREG-GFP and cathepsin L. In contrast, the subcellular distribution of dCREG-GFP did not overlap with that of Golgi-mannosidase II (Fig. 4). These findings clearly show that intracellular dCREG is an endosomal/lysosomal protein in S2 cells.

### 3.5 LERP depletion does not affect intracellular retention of dCREG by S2 cells

**Fig. 5:**
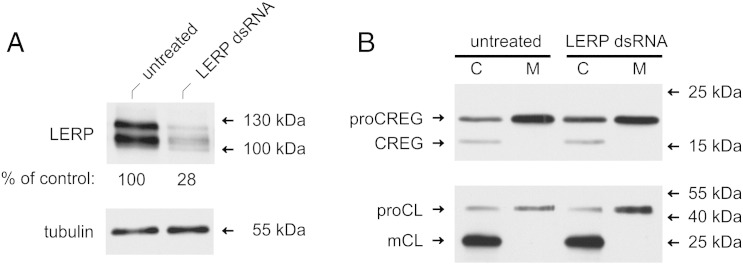
LERP is not critical for lysosomal sorting of dCREG. (A) S2-dCREG cells were treated with double-stranded (ds) LERP RNA for 72 h. Untreated cells cultivated under the same conditions were used as controls. Cell lysates (12 μg) were treated with PNGase F prior to immunoblotting with antibodies to LERP. Tubulin served as loading control. Band intensities were determined by densitometry. Data are expressed as normalized means of three independent experiments. (B) Cell lysates (C; 6 μg) and conditioned media (M) corresponding to 0.15 μg (dCREG) or 1.2 μg (cathepsin L) cellular protein were analyzed by immunoblotting with the indicated antibodies after enzymatic deglycosylation where appropriate (dCREG). proCREG, full-length dCREG; CREG, processed dCREG; proCL, procathepsin L; mCL, mature cathepsin L. The migration positions of selected molecular mass standards are indicated. The results shown are representative of three independent experiments.

In mammals, receptor-mediated sorting to lysosomes largely depends on the specific recognition of lysosomal proteins by M6P receptors. Recently, an M6P receptor homologue named lysosomal enzyme receptor protein (LERP) has been identified in *D. melanogaster*. Heterologous expression of LERP in M6P receptor-deficient murine embryonic fibroblasts led to a partial rescue of lysosomal enzyme misrouting [15]. When overexpressed in S2 cells, LERP was localized in *trans*-Golgi compartments and interacted with the clathrin adaptors GGA and AP-1 [29], [30]. However, the endogenous forms of this protein have not been characterized so far. For detection of endogenous LERP, a soluble form of the protein (amino acids 42–781) was produced in the baculovirus expression system and used to raise antibodies in rabbits. Immunoblotting analysis of total S2-dCREG extracts with antibodies to recombinant LERP revealed two bands of 135 and 115 kDa. Both bands were sensitive to PNGase F resulting in an increase of the respective electrophoretic mobility by approximately 10 kDa in either case. Knock-down of LERP caused loss of both signals, indicating the specificity of the antiserum (Fig. 5A).

To assess if LERP is involved in intracellular retention of dCREG, we performed knock-down experiments in S2-dCREG cells. The extent of LERP depletion achieved in these experiments amounted to 72 ± 1% (Fig. 5A). Consistent with the notion that LERP could contribute to the sorting of lysosomal enzymes in flies [15], retention of cathepsin L was moderately reduced in LERP-depleted cells (57 ± 1%) as compared to sham-treated controls (66 ± 1%; p = 0.002). Interestingly, maturation of dCREG was found to be slightly less effective when S2-dCREG cells were subjected to LERP-RNAi (27 ± 2% mature dCREG as compared to 32 ± 1% in the controls; p = 0.057). This could be due to the reduced cathepsin L content of LERP-depleted cells. However, the efficiency of dCREG retention (1.7 ± 0.3%) was not lower than in control cultures (1.0 ± 0.1%; Fig. 5B). We also investigated whether LERP could directly bind dCREG *in vitro*, using differentially tagged recombinant proteins. No specific interaction could be observed under any of the conditions tested (data not shown). These data suggest that LERP is dispensable for intracellular retention of dCREG, indicating that LERP is, unlike its mammalian relatives, not a universal sorting receptor for lysosomal proteins in flies.

### 3.6 Cathepsin L is required for turnover of dCREG in *D. melanogaster* flies

**Fig. 6:**
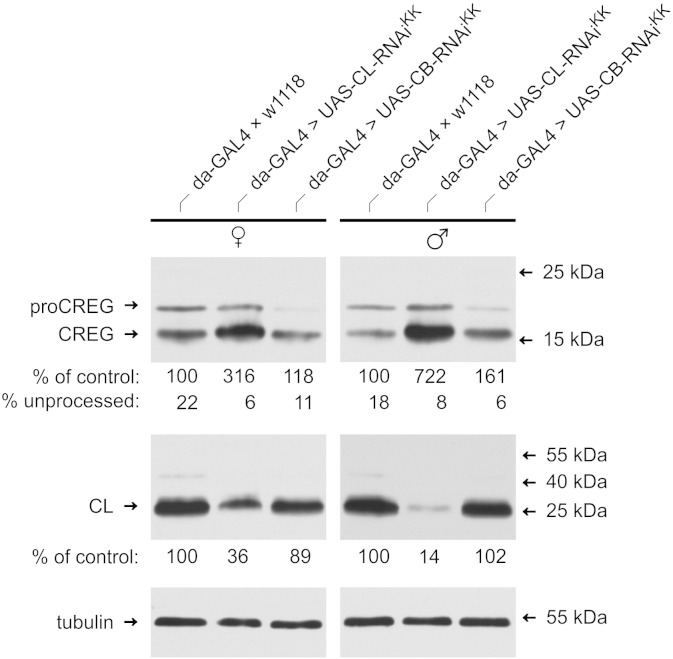
Cathepsin L knock-down impairs turnover of dCREG *in vivo*. Extracts from daughterless-GAL4 (da-GAL4) × w^1118^ (control), da-GAL4 > UAS-CB-RNAi^KK^ and da-GAL4 > UAS-CL-RNAi^KK^ flies were immunoblotted and probed with antibodies to dCREG (top), cathepsin L (middle) and tubulin as loading control (bottom). Band intensities were determined by densitometry. The cathepsin L and dCREG contents of the respective knock-down flies were calculated relative to control animals. Data are expressed as means of four independent experiments. The migration positions of selected molecular mass standards are indicated. proCREG, full-length dCREG; CREG, processed dCREG; CL, cathepsin L; CB, cathepsin B.

Using UAS-RNAi and GAL4-driver lines, we also investigated the consequences of depletion of cathepsins B or L on the processing status and the protein levels of dCREG in whole animals. UAS-cathepsin B-RNAi, UAS-cathepsin L-RNAi and the wild-type strain w^1118^ were crossed with the ubiquitous daughterless-GAL4 (da-GAL4) driver line. Extracts of hemizygous, freshly eclosed females and males were then analyzed by immunoblotting. Interestingly, knock-down of cathepsin L in male flies was more efficient (86 ± 6%) than in female flies (64 ± 16%) resulting in an increased total dCREG content of 722 ± 190% (males; p < 0.05) and 316 ± 53% (females; p < 0.01). In contrast, and consistent with our *in vitro* results (Fig. 2A), turnover of dCREG was much less affected in cathepsin B-depleted flies: dCREG levels amounted to 161 ± 19% (p < 0.05) in males and 118 ± 18% in females when compared to control (da-GAL4 × w^1118^) animals. However, dCREG processing was not compromised in the knock-down flies (Fig. 6). These results further emphasize that cathepsin L is the major proteinase in *D. melanogaster* responsible for the turnover of dCREG, with some contributions also by cathepsin B.

### 3.7 dCREG is essential for *D. melanogaster* development

**Fig. 7:**
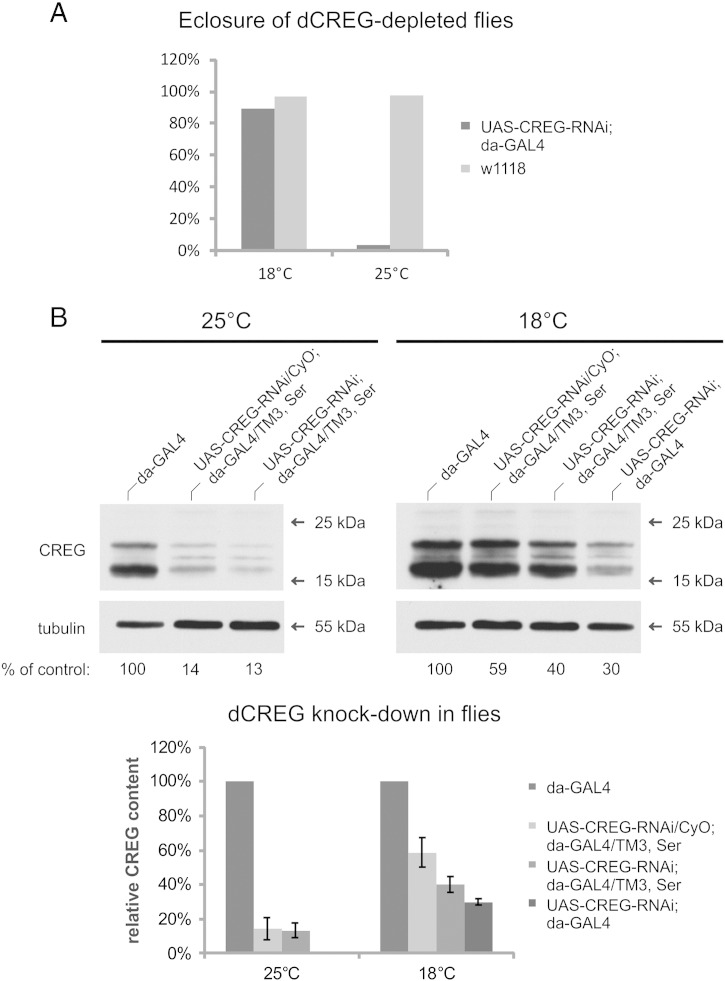
dCREG depletion in *D. melanogaster* flies. (A) Double homozygous UAS-CREG-RNAi; da-GAL4 and wild-type (w^1118^) flies were propagated at 18 °C or shifted to 25 °C and supplied with fresh media daily for 5 consecutive days. The percentage of eclosed flies was calculated. (B) CREG knock-down efficiency is dependent on temperature and gene dosage. Control (da-GAL4) and UAS-CREG-RNAi/CyO; da-GAL4/TM3, Ser flies were grown at 18 °C or 25 °C. Freshly eclosed flies were subjected to immunoblotting analysis with antibodies to dCREG and tubulin as loading control. Relative dCREG levels were determined by densitometry. Data are expressed as means of three independent experiments.

In order to create a stable dCREG knock-down fly line, UAS-CREG-RNAi and da-GAL4 lines were crossed with double balancers (If/CyO; Sb/TM3, Ser) to obtain UAS-CREG-RNAi; Sb/TM3, Ser and If/CyO; da-GAL4 flies. These were then crossed to generate UAS-CREG-RNAi/CyO; da-GAL4/TM3, Ser offspring. By crossing the latter with each other, we obtained flies homozygous for both UAS-CREG-RNAi and da-GAL4. We assumed that the most efficient dCREG knock-down would be achieved in such double homozygous flies. Moreover, we exploited the reduced activity of GAL4 at 18 °C [31] to maintain the double homozygous stock. To induce strong dCREG depletion, we shifted UAS-CREG-RNAi; da-GAL4 flies cultivated at 18 °C to 25 °C, resulting in lethality at late pupal stage (only 3% of all pupae eclosed). When control w^1118^ flies were shifted from 18 °C to 25 °C, 98% of all pupae eclosed. When grown at 18 °C, 89% of UAS-CREG-RNAi; da-GAL4 and 97% of w^1118^ flies eclosed (Fig. 7A).

To address whether dCREG knock-down was enhanced by increased incubation temperature and RNAi cassette/driver copy numbers, the relative dCREG content of the respective fly extracts was determined by immunoblotting. At 18 °C, the dCREG level in UAS-CREG-RNAi/CyO; da-GAL4/TM3, Ser (1 × RNAi, 1 × driver) amounted to 59 ± 9%, in UAS-CREG-RNAi; da-GAL4/TM3, Ser (2 × RNAi, 1 × driver) to 40 ± 4% and in UAS-CREG-RNAi; da-GAL4 (2 × RNAi, 2 × driver) to 30 ± 2%. When flies were reared at 25 °C, however, knock-down was much more efficient. The dCREG content was already reduced to 14 ± 7% in UAS-CREG-RNAi/CyO; da-GAL4/TM3, Ser flies, while in UAS-CREG-RNAi; da-GAL4/TM3, Ser flies the residual dCREG content was determined as 13 ± 4% (Fig. 7B). As already mentioned above, hardly any double homozygous flies could be obtained from crosses maintained at 25 °C. Thus, efficient dCREG depletion is necessary to cause lethality at the pupal stage.

**Fig. 8:**
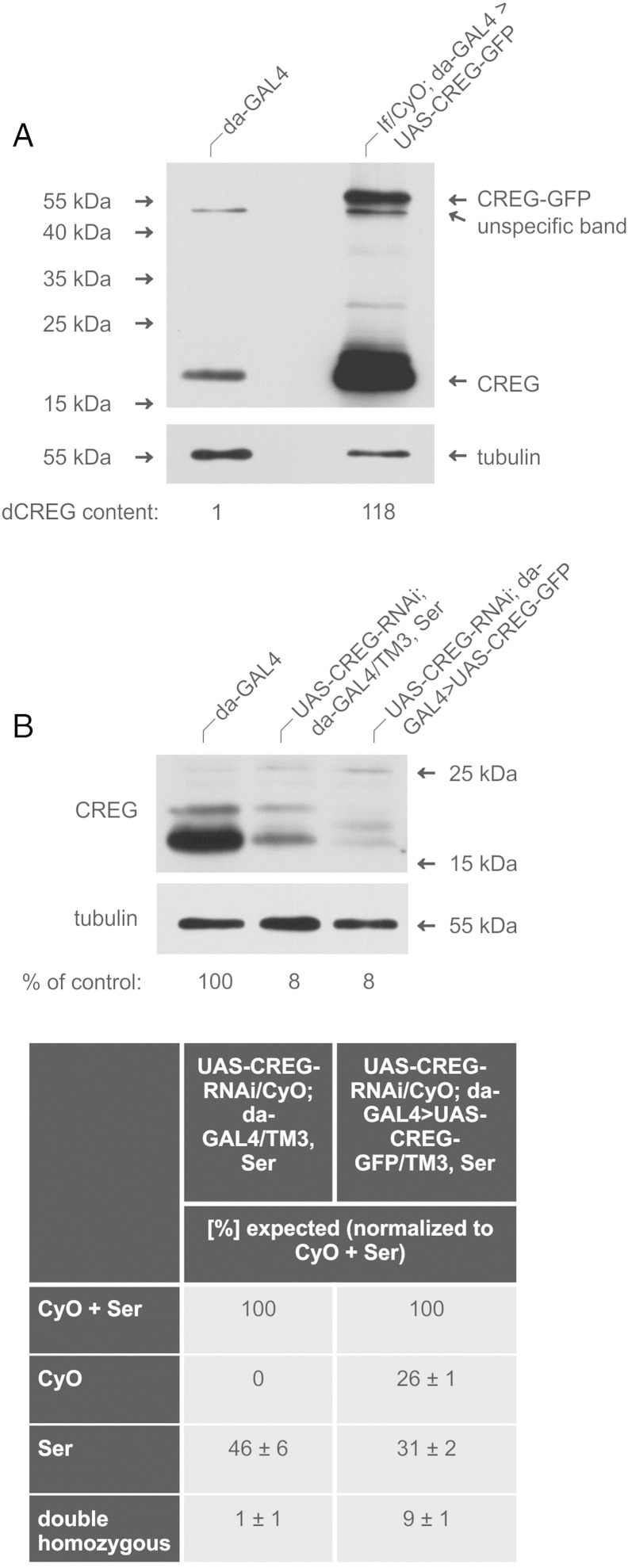
Rescue of dCREG knock-down flies by dCREG-GFP expression. (A) Lysates of da-GAL4 (control) and If/CyO; da-GAL4 > UAS-CREG-GFP flies were immunoblotted with antibodies to dCREG and tubulin as loading control. (B) Progeny of UAS-CREG-RNAi/CyO; da-GAL4/TM3, Ser and UAS-CREG-RNAi/CyO; da-GAL4 > UAS-CREG-GFP/TM3, Ser was counted (500–1000 flies per assay). Data from three independent experiments are expressed as means ± SE. Lysates from control, dCREG knock-down (UAS-CREG-RNAi; da-GAL4/TM3, Ser) and rescue flies (UAS-CREG-RNAi; da-GAL4 > UAS-CREG-GFP) flies were subjected to immunoblot analysis with antibodies to dCREG and tubulin as loading control.

In order to confirm that the observed lethality is not the consequence of off-target effects of the RNAi sequence used, we recombined a UAS-CREG-GFP construct with da-GAL4 on the third chromosome (If/CyO; da-GAL4 > UAS-CREG-GFP) and crossed the resulting line with UAS-CREG-RNAi; Sb/TM3, Ser flies. The expression of dCREG-GFP in these recombined flies was verified by immunoblotting, revealing that the majority of the dCREG-GFP fusion protein is cleaved into its constituents. This leads to a more than 100 times higher dCREG content of dCREG-GFP overexpressing flies as compared to da-GAL4 controls (Fig. 8A). Counting offspring of UAS-CREG-RNAi/CyO; da-GAL4/TM3, Ser and UAS-CREG-RNAi/CyO; da-GAL4 > UAS-CREG-GFP/TM3, Ser flies cultivated at 25 °C showed that simultaneous overexpression of dCREG-GFP cDNA partially rescued dCREG knock-down lethality, as demonstrated by an increase in surviving double homozygous animals from 1 ± 1% to 9 ± 1%. The successful rescue becomes even more evident when the number of flies carrying one copy of UAS-CREG-RNAi and two copies of the da-GAL4 driver is compared with that of the corresponding rescue flies (0% and 26 ± 1% of the expected respective offspring numbers). It should be pointed out that our dCREG-GFP construct is also a target of the dCREG-RNAi sequence used in this study, which probably explains why a full rescue could not be achieved. Notably, the dCREG content of the rescued flies corresponded only to 8% of that of da-GAL4 controls (Fig. 8B). Similar results were obtained with another, independently generated dCREG rescue line. Taken together, these results demonstrate that dCREG is essential for proper fly development once the level of this protein is reduced to below 8% of the normal amount. This compares well to lysosomal enzyme deficiencies in man, where restoration of 10–15% of the missing hydrolase activity is often sufficient to correct the respective clinical phenotype.

**Fig. 9:**
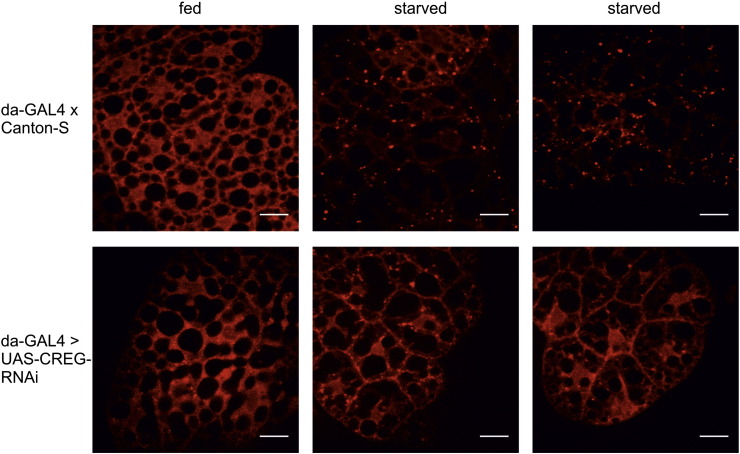
Reduced Lysotracker accumulation in fat bodies of dCREG knock-down flies. Control (da-GAL4 × Canton-S) and da-GAL4 > UAS-CREG-RNAi larvae were either starved for 4 h or further maintained on regular food (fed). Fat bodies were then removed and stained with Lysotracker Red prior to analysis by confocal laser-scanning microscopy. Bars, 20 μm.

**Fig. 10:**
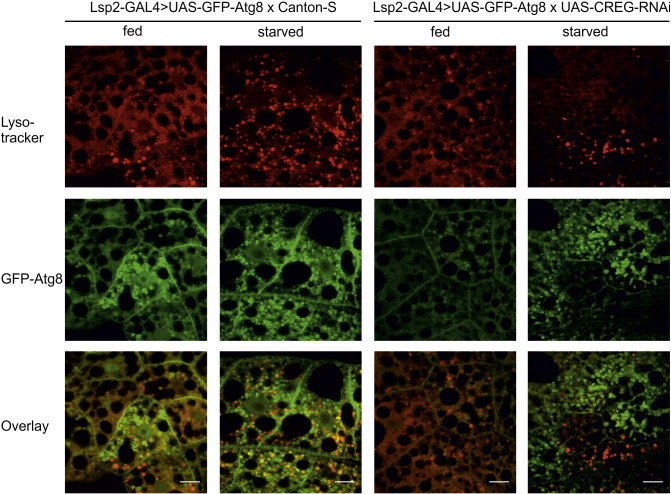
Autophagosome formation is not impaired in dCREG knock-down flies. Control (Lsp2-GAL4-UAS-GFP-Atg8 × Canton-S) and Lsp2-GAL4-UAS-GFP-Atg8 × UAS-CREG-RNAi larvae were either starved for 4 h or further maintained on regular food (fed). Fat bodies were then removed and stained with Lysotracker Red. Acidic compartments (Lysotracker) and autophagosomes (GFP-Atg8) were detected by confocal laser-scanning microscopy. Co-localization was assessed by merging of the individual images. Bars, 20 μm.

In *D. melanogaster*, the functionality of the lysosomal compartment is studied best in the fat bodies of third-instar larvae (the developmental stage preceding pupation). In these organs, the starvation-induced generation of autolysosomes is readily detectable by live-cell imaging with Lysotracker Red [23], [32]. While pronounced dye accumulation was observed in most control samples (82%), the fraction of Lysotracker-positive da-GAL4 > UAS-CREG-RNAi sections (31%) was substantially smaller (Fig. 9). Similar results were obtained when a fat body-specific GAL4 driver (Lsp2) was used. Whereas the vast majority of wild-type tissues displayed strong Lysotracker staining (88%), such a response was only seen in less than half (43%) of the dCREG-depleted specimens. However, sequestration of GFP-Atg8 into punctate structures was similar in Lsp2-GAL4-UAS-GFP-Atg8 × UAS-CREG-RNAi and control fat bodies, demonstrating that dCREG knock-down does not impair autophagosome formation (Fig. 10). Moreover, the median survival time of starved da-GAL4 > UAS-CREG-RNAi larvae (42 h) was the same as that of control animals. Since disruption of autophagy genes is known to cause hypersensitivity to starvation in *D. melanogaster* [32], these results indicate that dCREG depletion does not interfere with the induction of a robust autophagic response.

## 4 Discussion

This report demonstrates that dCREG is an endosomal/lysosomal glycoprotein, which undergoes proteolytic maturation during its biosynthesis similar to its mammalian counterparts. Our previous study showed that murine CREG co-localizes with the lysosomal enzymes cathepsin B and cathepsin D in fibroblasts [10]. To determine the subcellular localization of dCREG in S2 cells, we first showed substantial co-localization between dCREG-GFP and a lysosomotropic dye by live-cell microscopy. We have also observed co-localization between dCREG and cathepsin L, a lysosomal protein in *D. melanogaster* cells [33].

Mammalian CREG undergoes limited processing upon delivery to lysosomes. Our previous study revealed that inhibition of lysosomal cysteine cathepsins in murine fibroblasts leads to accumulation of a processing intermediate, but the proteinases involved in this maturation event could not be identified [10]. In humans, 11 cysteine cathepsins have been found (cathepsins B, C, F, H, K, L, O, S, V, W and Z). Cathepsins F, K, L, S and V appear to be obligate endopeptidases, whereas cathepsins B and H display exopeptidase as well as endopeptidase activities. Two genuine exopeptidases also belong to this family, the dipeptidyl aminopeptidase cathepsin C and the carboxypeptidase cathepsin Z [28]. Four of these enzymes (cathepsins B, F, K and L) have been also detected in *D. melanogaster* [27]. We have now observed that cathepsin L is capable of processing dCREG both *in vitro* and *in vivo*. Importantly, recombinant dCREG digested with cathepsin L co-migrated with the mature polypeptide present in S2 extracts. N-terminal sequencing of dCREG treated with cathepsin L revealed the removal of the first 14 amino acids. A similar processing event has been described for human monocytes, where also a truncated form of CREG lacking its 14 N-terminal amino acids has been detected [25]. Further support for the involvement of cathepsin L in dCREG maturation was obtained by RNAi-mediated downregulation of the enzyme. Knock-down of this cysteine proteinase led to enhanced accumulation and partially impaired processing of dCREG in S2 cells. Moreover, reduced turnover of dCREG could also be observed upon cathepsin L depletion in whole flies. These results strongly support the notion that proteolytic cleavage of dCREG takes place after reaching endosomal/lysosomal compartments due to an endoproteolytic cleavage event which can be executed by cathepsin L. As demonstrated for cathepsin B, other lysosomal proteinases are probably also capable of removing the dCREG propeptide, thus explaining why dCREG processing is only partially impaired upon almost quantitative depletion of cathepsin L.

Previous studies have shown that mammalian CREG interacts with both M6P receptors, M6P/IGF2R and MPR46 in an M6P-dependent manner [8], [9], [10]. Our studies clearly show that intracellular retention of dCREG does not rely on *N*-glycosylation of the protein, indicating that carbohydrate–protein interactions are not involved in its delivery to lysosomes. Only very few protein-based lysosomal sorting signals have been identified so far, most of them in plants [34]. However, a recent study reported a carbohydrate-independent mode of action for the binding of human CREG to M6P/IGF2R domains 11–15, a fragment of the receptor which does not harbor a functional M6P-binding site [35]. Sequence alignments have revealed that its *D. melanogaster* relative LERP is homologous to receptor domains 9–13 [15]. This led us to test whether dCREG could bind to LERP via protein–protein interactions. However, our pull-down experiments did not provide any evidence for LERP being capable of directly associating with dCREG. Moreover, downregulation of LERP did not impede lysosomal sorting of dCREG in S2 cells, indicating that this putative lysosomal sorting receptor is not engaged in the delivery of dCREG to its intracellular destination. Noteworthy, our knock-down studies confirmed that LERP depletion leads to reduced intracellular retention of cathepsin L and thus reduced lysosomal cysteine proteinase activity as previously reported [16]. This finding provides a potential explanation for the slightly impaired proteolytic processing of intracellular dCREG upon LERP depletion in S2 cells, since cathepsin L appears to be involved in this maturation step.

In order to learn more about the physiological role of dCREG in *D. melanogaster*, we decided to investigate the consequences of dCREG depletion in this genetically tractable model organism. We could demonstrate that dCREG knock-down is lethal at late pupal stages. Importantly, rescue experiments confirmed that the observed pupal lethality is indeed due to dCREG depletion. Interestingly, manifestation of this phenotype appears to be associated with changes to the autolysosomal compartment. Although CREG has not yet been implicated in autophagy, recent studies have linked this protein to many other physiological processes such as cell migration [36], [37], replicative senescence [38], apoptosis [39], cell cycle progression [35] and proliferation [40]. However, a unifying concept which reconciles all these proposed functions of CREG has not been put forward so far. The interpretation of the available data on the putative cellular activities of CREG is also complicated by seemingly contradictory results reported in different studies. In smooth muscle cells, CREG has been found to inhibit migration [36] and to induce cell cycle arrest [35]. The opposite has been observed for endothelial cells, where overexpression of CREG led to enhanced mobility [37] and accelerated proliferation [40]. In this context, it is also of note that we were unable to confirm the anti-apoptotic potential of CREG reported for smooth muscle and mesenchymal stem cells [6], [7], [39] in other cellular systems (E. Kowalewski-Nimmerfall and L. Mach, unpublished results). Hence, the molecular mechanism accounting for the pupal lethality induced by CREG ablation in *D. melanogaster* is still to be elucidated.

This study was the first to assess the role of CREG in a living animal. To our knowledge, this is also the first report demonstrating that downregulation of a luminal lysosomal protein can cause developmental lethality in *D. melanogaster*. It is worth pointing out that the information available on the consequences of depleting individual lysosomal enzymes in flies is still scarce. So far, such studies have been only performed for a small number of acid hydrolases. Ablation of the cathepsin L gene has been shown to result in female sterility. Attenuated pigmentation of some abdominal segments was also observed. Other visible morphological effects include the appearance of the wings, which often contain bubbles and frequently fail to expand fully [41]. Inactivation of cathepsin D in flies results in age-dependent neurodegeneration and recapitulates the key features of neuronal ceroid lipofuscinoses, a family of lysosomal storage disorders [42]. Genetic analysis in *D. melanogaster* also revealed that cathepsin D plays a neuroprotective role by counteracting tau-induced neurotoxicity [43]. Similarly, mutations in the FDL gene encoding an endosomal/lysosomal β-*N*-acetylglucosaminidase affect normal development of the fly brain, resulting in major anatomical changes to the mushroom bodies as manifested by fused lobe structures [44]. Having established that dCREG is essential for proper completion of fly development, our results set the stage for *D. melanogaster* becoming the model organism of choice for future studies on the physiological functions of this lysosomal protein.
